# Thermal Hazard Evaluation of Tert-Butyl Peroxy-3,5,5-trimethylhexanoate (TBPTMH) Mixed with Acid-Alkali

**DOI:** 10.3390/ma15124281

**Published:** 2022-06-17

**Authors:** Li Xia, Lei Ni, Yong Pan, Xin Zhang, Yuqing Ni

**Affiliations:** 1 College of Safety Science and Engineering, Nanjing Tech University, Nanjing 211816, China; xiali@njtech.edu.cn (L.X.); xinzhang@njtech.edu.cn (X.Z.); nyqforwork@outlook.com (Y.N.); 2 Jiangsu Key Laboratory of Hazardous Chemicals Safety and Control, Nanjing 211816, China

**Keywords:** TBPTMH, acid-alkali, thermal decomposition, thermodynamic analysis, characteristic parameter

## Abstract

Tert-butyl peroxy-3,5,5-trimethylhexanoate (TBPTMH), a liquid ester organic peroxide, is commonly used as an initiator for polymerization reactions. During the production process, TBPTMH may be exposed to acids and alkali, which may have different effects on its thermal hazard, so it is necessary to carry out a study on the thermal hazard of TBPTMH mixed with acids and alkali. In this paper, the effects of H_2_SO_4_ and NaOH on the thermal decomposition of TBPTMH were investigated by differential scanning calorimetry (DSC) and adiabatic calorimetry (Phi-TEC II). The “kinetic triple factors” were calculated by thermodynamic analysis. The results show that the three E_a_ are 132.49, 116.36, and 118.24 kJ/mol, respectively; thus, the addition of H_2_SO_4_ and NaOH increased the thermal hazard of TBPTMH. In addition, the characteristic parameters (time to maximum rate under adiabatic conditions, self-accelerated decomposition temperature) of its thermal decomposition were determined, and the control temperature (45, 40, and 40 °C) of TBPTMH under the action of acid-alkali were further received. This work is expected to provide some guidance for the safe storage, handling, production, and transportation of TBPTMH in the process industry.

## 1. Introduction

Organic peroxides are substances produced by replacing hydrogen atoms in hydrogen peroxide with organic groups such as alkyl, acyl, and aromatic groups [1,2]. It is a pivotal chemical raw material in industrial production. Its general formula is R1-O-O-R2. However, the O-O bond is relatively weak, making organic peroxides extremely unstable and likely to decompose due to external factors such as high temperature, friction, and impact [3,4]. If the heat generated by thermal decomposition is not removed in time, it will trigger a number of uncontrollable chain reactions, eventually leading to fire and explosion accidents.

**Figure 1:**
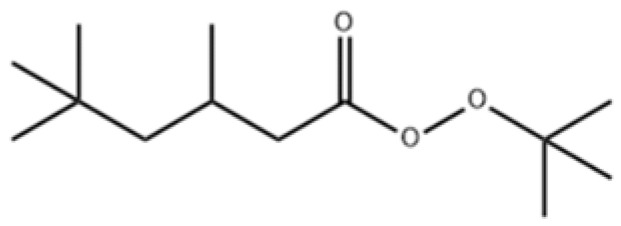
Chemical structural formula of TBPTMH.

TBPTMH is a liquid ester organic peroxide, and its structural formula is shown in Figure 1. It belongs to a chemical fragrance and flavor intermediate, which is commonly used as an initiator of polymerization reactions (such as ethylene, styrene, methyl methacrylate, allyl compounds). It is safer than tert-butyl peroxybenzoate as an initiator for styrene polymerization [5]. During the production process, acid and alkali may be added to catalyze the reaction, and during usage, it may cause thermal runaway [6].

Recently, a number of studies have paid attention to TBPTMH and organic peroxides with acid-alkali. A brief overview of the literature is shown in Table 1. Yang [7] et al. used differential scanning calorimetry (DSC) to investigate the thermal hazard of TBPTMH at the heating rate of 0.5/1/2/4 °C/min and fitted kinetic equations to calculate the self-accelerated decomposition temperature (SADT) at 10, 25, 35, and 50 kg packing mass. The study revealed that the initial decomposition temperature of TBPTMH was 103 °C, and the heat release was as high as 924 J/g. The SADT values decreased with increasing mass, so the storage of TBPTMH should be given extra attention, which is immensely dangerous in case of decomposition. Chen [5] et al. employed DSC to explore the thermal risk of TBPTMH in the presence of BPO. The results showed that the initial temperature (T_0_) of TBPTMH+BPO was higher than that of TBPB+BPO, but the heat release was lower. Therefore, it is safer to apply TBPTMH as an initiator than TBPB in the production of molding compounds. Tseng et al. [8] used VSP2 and DSC to investigate the effect of adding HCl (6 N), HNO_3_ (6 N), H_3_PO_4_ (6 N), and H_2_SO_4_ on MEKPO. The study showed that MEKPO is very sensitive to acid, especially HNO_3_ at 6 N. The addition of acid in both adiabatic and open environments decreased its T_0_. Liu [2] et al. studied the effect of H_2_SO_4_, NaOH, Na_2_SO_3_ on CHP, BPO, and DCPO using DSC, TAM III, and VSP2. The results showed that the risk of CHP, BPO, and DCPO was significantly increased by the addition of the above influences. You [9] et al. used DSC to investigate the effect of adding different concentrations of HNO_3_ on the thermal hazard of lauryl peroxide (LPO) and to calculate its TMR and SADT. It was found that LPO was very sensitive to inorganic acids, and its hazard increased with the increase in HNO_3_ concentration. Moreover, after the addition of HNO_3_, two exothermic peaks of LPO were detected, and explosive 1-nitrododecane was formed in the product, which increased the danger of LPO significantly.

The above studies show that the thermal hazard of TBPTMH is high and is increased by contact between organic peroxides and acids and bases, so it is essential to study the effect of acids and bases on the thermal hazard of TBPTMH.

In this paper, the effect of acid-alkali on the thermal hazard of TBPTMH was studied by adding H_2_SO_4_ and NaOH. DSC and adiabatic calorimetry (Phi-TEC II) were used to analyze their thermal hazards. The thermodynamic analysis was performed using thermodynamic theory to acquire the thermodynamic parameters, and the time to maximum rate under adiabatic conditions (TMR_ad_), self-accelerated decomposition temperature (SADT), and reaction mechanism functions were further determined. This work investigates the effect of added acids-alkali on TBPTMH. Its decomposition in the adiabatic state and the mechanism function are also investigated, which is the innovative point of this paper. These findings are expected to provide guidance for the safe storage, transportation, and production of TBPTMH.

## 2. Materials and Methods

### 2.1. Sample Preparation

**Figure 2:**
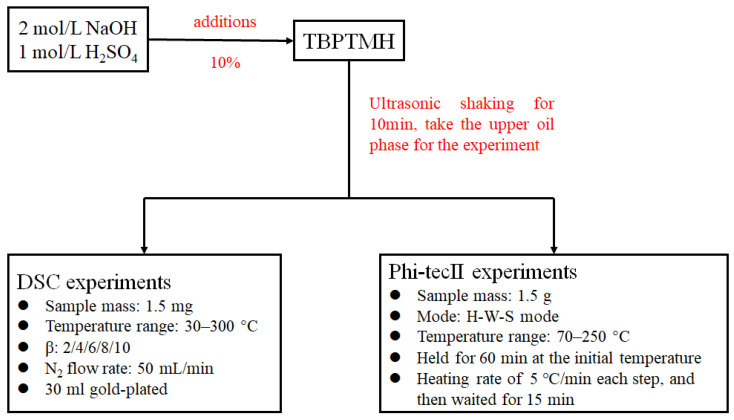
The experimental flow chart.

TBPTMH used in this study was purchased from Wengjiang Reagent. H_2_SO_4_ and NaOH were obtained from Shanghai Lingfeng (Shanghai, China), and the purity of the above samples was higher than 98%. H_2_SO_4_ and NaOH were prepared at molar concentrations of 1 mol/L and 2 mol/L, respectively. The addition amounts of both influencing factors were 10%, and the upper oil phase was shaken by ultrasonication for 10 min and left to take for the experiment. The experimental flow chart is shown in Figure 2.

### 2.2. Differential Scanning Calorimetry

Differential scanning calorimetry (DSC) is a technique that measures the variation in energy or power difference between the sample and the reference with temperature, which is controlled by a program [10,11]. It can explore the physical or chemical changes of a substance during heating or cooling and is generally a frequently used method for thermal analysis. It is widely used in the fields of rubber, inorganic materials, biological organisms, metallic materials, and composite materials [12]. It has the characteristics of high sensitivity, high resolution, reliable test data, and low sample consumption.

In this paper, the HP DSC 1 with low inertia and fast temperature rise and fall made by Mettler Toledo was used in experiments. The double safety of an explosion-proof sheet and sealing system made the experiment safer. The temperature range was 30–300 °C under a nitrogen environment with a flow rate of 50 mL/min. Of gold-plated crucible, 30 μL filled with 1.5 mg of sample was applied for each experiment.

### 2.3. Adiabatic Calorimetry

The characteristic parameters of a sample measured by DSC vary with the heating rate, so the results of DSC experiments can only be adopted for preliminary investigation of the thermal decomposition properties of the sample. The Phi-TEC II can detect the weak exothermic signal of the sample under adiabatic conditions to improve the accuracy of the results, which is one of the most commonly used apparatuses for thermal analysis. It can obtain the temperature and pressure curves with time during sample decomposition in an adiabatic environment [13,14].

In this paper, an adiabatic calorimetry (Phi-TEC II) manufactured by HLE was performed in the H-W-S (heat-wait-search) mode [15,16]. Phi-TEC II detects the temperature by direct contact of a thermocouple with the sample, which can improve the detection sensitivity of the exotherm of the tested sample. In this section, a sample of about 1.5 g was used, and the material of the spheres employed was Hastelloy. The test temperature range was 70–250 °C. The program held for 60 min at the initial temperature, heated at a heating rate of 5 °C/min each step, and then waited for 15 min. When the program detected an exothermic rate of more than 0.02 °C/min, it turned into the adiabatic tracking mode. The maximum bearable pressure is 70 bar.

### 2.4. Thermodynamic Analysis

In chemical processes, thermodynamic analysis can be used as an important indicator parameter to determine optimal process conditions. In recent years, as the demand for chemical processes and the maturity of experimental techniques for thermal analysis have increased, this method has commonly been applied to evaluate the thermal stability of organics, inorganics, and polymers. Thermodynamic analysis is mainly used to obtain the “kinetic triple factors” (pre-exponential factor (A), activation energy (E_a_), and reaction mechanism function) of a reaction by means of established mechanism functions [17,18,19].

#### 2.4.1. Starink Method

The Starink method was deduced by analyzing the summary of the errors existing in the KAS, FWO, and Boswell methods. The E_a_ can be found by the Starink method, as shown in Equation (1). Compared with the KAS and FWO methods, the activation energy calculated by it has higher accuracy, which is recommended by ICTAC and is widely used in fine chemical calculations [20,21,22,23].

(1) $$\ln\left(\frac{\beta }{T^{1.92}}\right)=-1.0008\frac{E_{a}}{\mathrm{RT}}+\mathrm{const}$$

where β is the heating rate, E_a_ is the activation energy, R is the gas constant, and T is the temperature

#### 2.4.2. Correction of Adiabatic Data

The ideal adiabatic environment is one in which the environment does not absorb the energy generated by the exotherm of the sample. However, in the actual Phi-TEC II experiments, the heat released by the sample is used to heat not only the sample itself, but also the ambient temperature of the cuvette. Therefore, Phi-TEC II does not fully satisfy the adiabatic condition either. The acquired adiabatic temperature rise and other parameters cannot be directly used to characterize the sample itself, and a thermal inertia factor must be introduced to correct the experimental data, as shown in Equation (2) [24,25,26]:

(2) $$\emptyset =1+\frac{M_{b}C_{\mathrm{vb}}}{M_{s}C_{\mathrm{vs}}}$$

where M_b_ and M_s_ are the mass of the sample ball and sample, respectively. C_vb_ and C_vs_ represent the specific heat capacity of the sample ball and sample.

#### 2.4.3. Adiabatic Dynamics Calculation

In an adiabatic environment, the exothermic decomposition process of the sample is in accordance with the Arrhenius equation, as shown in Equation (3) [27]:

(3) $$\mathrm{lnk}=-\frac{E_{a}}{\mathrm{RT}}+\mathrm{lnA}$$

By choosing a different reaction order, the graph of lnk−1/T was plotted. Substituting the experimental data, a series of points at a different reaction order can be calculated. These points were fitted and analyzed, and the best-fitting line was selected, from which the activation energy and pre-exponential factor can be further calculated.

#### 2.4.4. Thermal Decomposition Reaction Mechanism Function

The Coats–Redfern method solves for the reaction activation energy by means of a mechanism function, which is usually used to probe the thermal hazard of a substance in depth, as shown in Equation (4) [28,29,30,31]. In this paper, the Coats–Redfern method is used to investigate the mechanism function of TBPTMH and the addition of H_2_SO_4_ and NaOH, and to guide the determination of the mechanism function of the sample by the reaction level obtained.

(4) $$\ln\frac{G\left(\alpha \right)}{T^{2}}=-\frac{E_{a}}{\mathrm{RT}}+\ln\frac{\mathrm{AR}}{{\beta E}_{a}}$$

where G(α) denotes the mechanism function, and the commonly used mechanism functions are summarized in Table 2.

### 2.5. Calculation of Thermal Decomposition Characteristic Parameters

#### 2.5.1. Calculation of TMR_ad_

TMR_ad_ is the time to maximum reaction rate under adiabatic conditions, which is also known as the adiabatic induction period, as shown in Equation (5) [32]. TMR_ad_ can be used to describe the time to take protective measures when a decomposition reaction is runaway, which is usually considered to be proportional to the length of time to take protective measures. In chemical processes, we usually focus on the temperature corresponding to TMR_ad_ = 8 h and TMR_ad_ = 24 h.

(5) $${\mathrm{TMR}}_{\mathrm{ad}}=\frac{{\mathrm{RT}}^{2}}{{\mathrm{AE}}_{a}\Delta T_{\mathrm{ad}}C^{n-1}{\left(\frac{T_{f}-T}{\Delta T_{\mathrm{ad}}}\right)}^{n}\exp\left(-\frac{E_{a}}{\mathrm{RT}}\right)}$$

where ∆T_ad_ is the adiabatic temperature rise, C is the specific heat capacity of the sample, and T_f_ is the maximum decomposition temperature under adiabatic conditions.

#### 2.5.2. Calculation of SADT

SADT is the lowest temperature at which self-accelerated decomposition of a chemical substance using packaging occurs [33,34], which can characterize the critical temperature of an uncontrolled reaction, so it can be used to evaluate the thermal hazard of chemicals under real situations, as shown in Equation (7).

(6) $$T_{\mathrm{NR}}=\frac{A\Delta {\mathrm{HM}}^{n}E_{a}}{\mathrm{RUS}}\exp\left(-\frac{E_{a}}{{\mathrm{RT}}_{\mathrm{NR}}}\right)$$

where T_NR_ is the non-return temperature, M is the mass of the drug package, R is the gas constant, U is the heat transfer coefficient of the packaging material, and S is the surface area of the package.

(7) $$\mathrm{SADT}=T_{\mathrm{NR}}-\frac{T_{\mathrm{NR}}^{2}}{E_{a}}$$

During actual production, transportation, and storage, if the ambient temperature is higher than the value of SADT, the chemical will incur self-decomposition. The heat generated by the reaction is used to heat the reaction system, resulting in an increase in external temperature, which further promotes the decomposition of the chemical. Therefore, the ambient temperature of the chemical should be lower than the value of SADT, the temperature of the chemical should be recorded, and emergency rescue countermeasures should be formulated to prevent the reaction from occurring and causing more damage.

## 3. Results and Discussion

### 3.1. DSC Experiments

**Figure 3:**
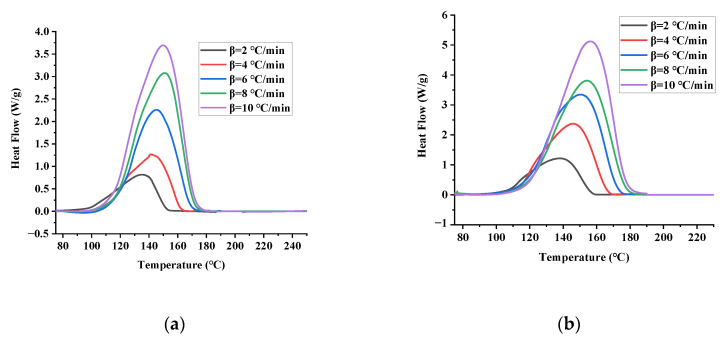

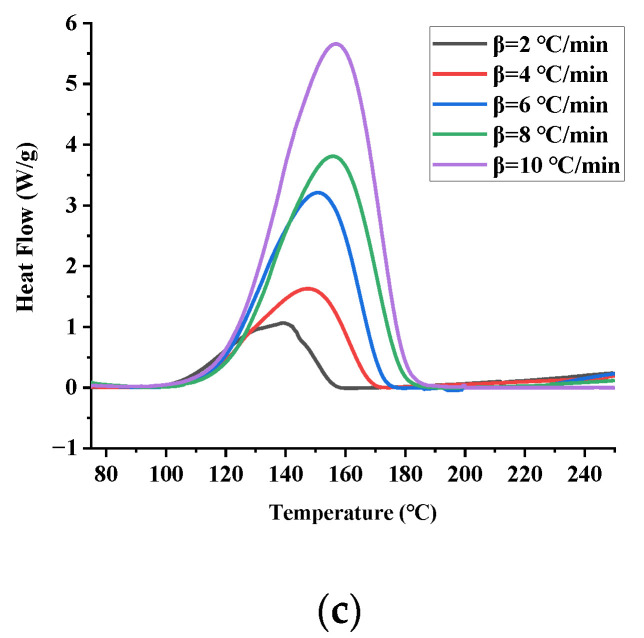
Heat flow curves of pure TBPTMH (**a**) and mixtures with H_2_SO_4_ and NaOH (**b**,**c**) at different heating rates by DSC tests.

Heat flow curves of pure TBPTMH and under the action of H_2_SO_4_ and NaOH are shown in Figure 3a–c; the characteristic parameter values are depicted in Table 3.

As can be seen in Figure 3a, there is only one exothermic peak of TBPTMH, indicating that the thermal decomposition of TBPTMH is a one-step reaction. The peak height and width increase with the increase in the heating rate, and the peak shape becomes sharper. This phenomenon occurs because the sensitivity of the instrument decreases with the increase in the heating rate. The average values of T_0_, T_p,_ and ΔH of TBPTMH thermal decomposition are 118.31 °C, 150.38 °C, and 687.42 J/g. Therefore, if TBPTMH decomposes and accumulates heat, a runaway reaction can easily occur.

As can be seen in Figure 3b,c, the addition of H_2_SO_4_ and NaOH remains as a single decomposition exothermic peak and does not change the decomposition peak of TBPTMH. According to the average of the data obtained from Table 3, the T_0_ of adding H_2_SO_4_ and NaOH are 112.54 and 117.86 °C, respectively. It can be seen that the addition of H_2_SO_4_ and NaOH reduced the T_0_ of TBPTMH and made TBPTMH more decomposable, with a peak temperature (T_p_) of 148.88 and 149.74 °C and ΔH of 751.49 and 686.44 J/g. Compared with the ΔH of TBPTMH, it was found that the addition of acid-alkali made it higher, which indicated that more heat was generated, and the decomposition was more dangerous. Therefore, special attention should be paid to avoid contact with acid-alkali during the production, transportation, and storage of TBPTMH.

### 3.2. Phi-TEC II Experiments

**Figure 4:**
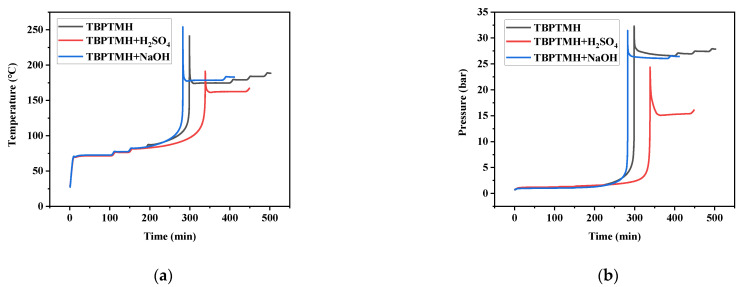
Temperature–time curve (**a**) and pressure–time curve (**b**).

The temperature–time curves(a) and pressure–time curves(b) of TBPTMH and the addition of H_2_SO_4_ and NaOH under adiabatic environment are illustrated in Figure 4, and the characteristic experimental values are listed in Table 4. In Figure 4, it can be seen that the exotherm and the temperature rise are not conspicuous at the beginning of decomposition. With the progress in time, the heat accumulation accelerates the reaction, the temperature increases steeply, and the exothermic rate also increases obviously, showing a sharp peak shape. The change trend of pressure is similar to temperature.

In Figure 4, it can be seen that TBPTMH starts to exotherm at 87.68 °C, and the temperature rises extremely after 100 °C, reaching a maximum temperature rise rate of 167.9 °C/min at 239.26 °C, and the pressure can reach a maximum of 32.3 bar. Therefore, once the decomposition reaction of TBPTMH occurs, it may cause serious consequences. Further, T_0_ after adding H_2_SO_4_ and NaOH is 81.58 and 83.2 °C, respectively. Compared with pure TBPTMH, their T_0_ was reduced, which was consistent with the conclusions of previous DSC experiments. ∆T_ad_ rose particularly significantly with the addition of NaOH, close to about 20 °C, and the maximum temperature rise rate ((dT/dt)max)) and maximum pressure rise rates ((dP/dt)max)) were 192.85 °C/min and 168.36 bar/min, respectively, which should obviously indicate extra caution about the contact of NaOH with TBPTMH.

### 3.3. Thermodynamic Analysis

#### 3.3.1. Starink Method

**Figure 5:**
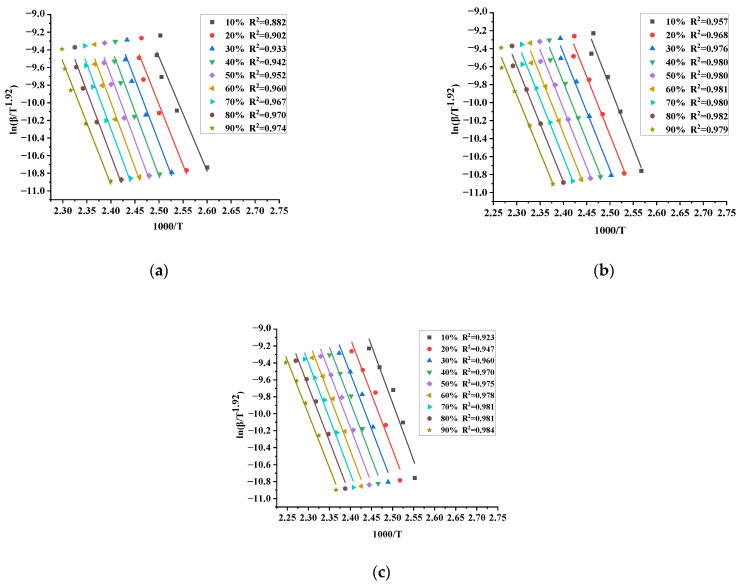
Starink method fitting curve of TBPTMH (**a**) and mixtures with H_2_SO_4_ and NaOH (**b**,**c**).

MATLAB software was used to fit the experimental data according to the least-squares method. To ensure the accuracy of the results, the DSC experimental data with a conversion (α) range of 10–90% and an interval of 10% each time were selected. Finally, the activation energy could be determined according to the slope of each fitting line, and the fitting results are outlined in Figure 5a–c.

The activation energies corresponding to different α are specified in Table 5. It was found that the value of activation energy does not vary markedly at different α. Therefore, it can be inferred that TBPTMH has only one exothermic peak and is a single-step decomposition reaction, which is consistent with the conclusion reached in DSC experiments.

According to Table 5, the activation energies of TBPTMH and its added acid-alcali are 132.49 kJ/mol, 116.36 and 118.24 kJ/mol, respectively. The comparison shows that the activation energy relationship is: TBPTMH > TBPTMH + NaOH > TBPTMH + H_2_SO_4_. In other words, the effect extent of the influencing factors is: NaOH < H_2_SO_4_.

#### 3.3.2. Correction of Adiabatic Data

The adiabatic data were corrected according to Equation (2), and the corrected results are shown in Table 6. As can be seen in Table 6, the corrected ΔH, ΔT_ad_, dT/dt_max,_ and dP/dt_max_ of pure TBPTMH are all increased substantially, but T_0_ is decreased. This indicates that the decomposition of TBPTMH is more likely to occur under adiabatic conditions, and the decomposition rate is faster, which can lead to serious accidents in a short period of time.

When the TBPTMH with the addition of H_2_SO_4_ and NaOH was in an ideal adiabatic environment, ΔH and ΔT_ad_ were increased in magnitude. In comparison, it was found that the two influencing factors of adding H_2_SO_4_ and NaOH could increase the thermal hazard of TBPTMH, especially for the NaOH. ΔT_ad_ was as high as 720.69 °C. dT/dt_max_ reached 813.83 °C/min after adding NaOH, which indicated that if the decomposition reaction of TBPTMH occurs after contacting with NaOH, the reaction will become violent, and the temperature and pressure will increase sharply, resulting in a short time to take safety measures, making evacuation and emergency more difficult to achieve.

#### 3.3.3. Adiabatic Kinetic Calculation

**Figure 6:**
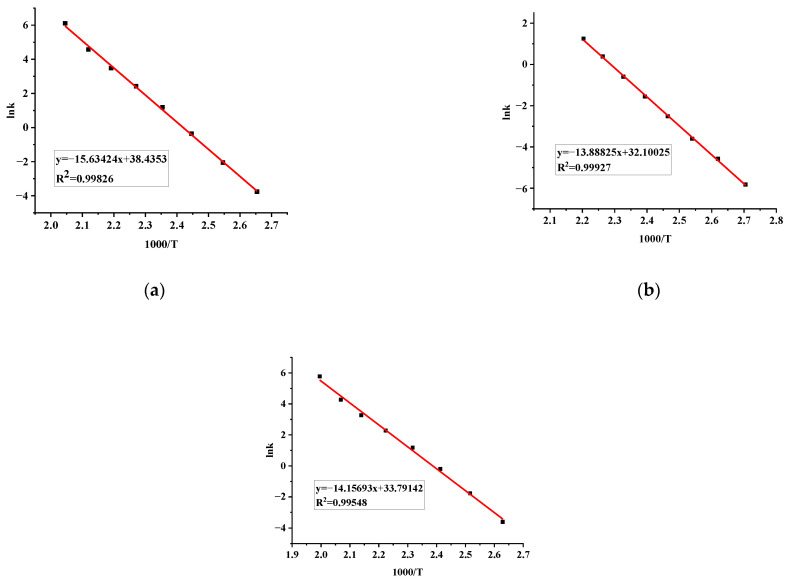
Adiabatic kinetic fitting results of pure TBPTMH (**a**) and mixtures with H_2_SO_4_ and NaOH (**b**,**c**).

The best-fitting straight line was selected by choosing a different reaction order (n) and fitting lnk-1/T, and the fitting results are disclosed in Figure 6a–c.

Based on the fitting Equation (3), the adiabatic kinetic calculation results are enumerated in Table 7. E_a_ for TBPTMH and added H_2_SO_4_ and NaOH were 129.98, 115.47, and 117.70 kJ/mol, respectively, and n were 2.6, 1, and 2.3, severally. The activation energies calculated by the Starink method were slightly lower than that in the adiabatic environment. This indicates that accidents are more likely to occur under adiabatic conditions than in open space. All the fitting coefficients are above 0.995, indicating that the results are accurate.

#### 3.3.4. Thermal Decomposition Reaction Mechanism Function

**Figure 7:**
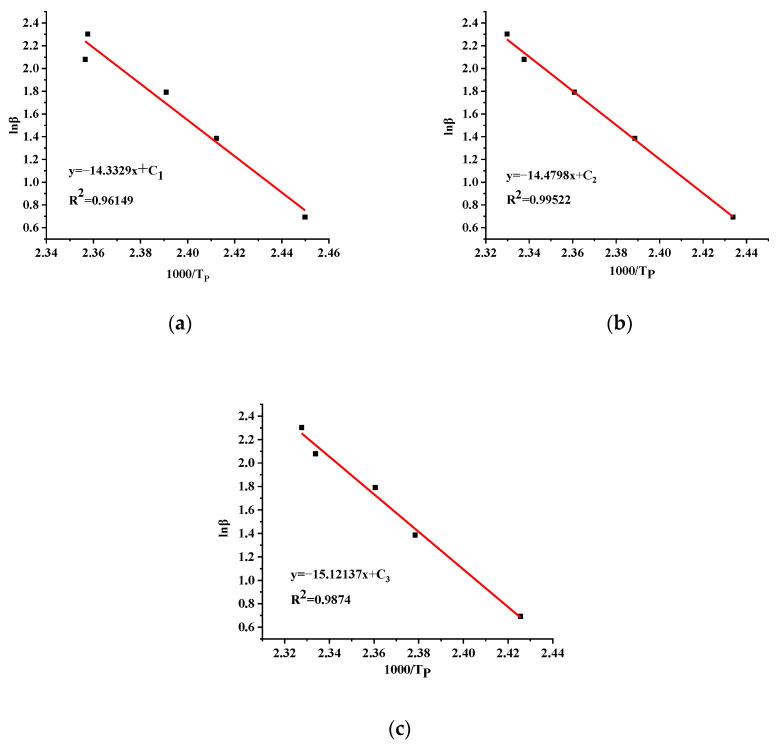
Fitting curves of pure TBPTMH (**a**) and mixtures with H_2_SO_4_ and NaOH (**b**,**c**) by Coats–Redfern method.

According to Equation (4), a plot is made for 1/T_P_-lnβ, and n is calculated from the slope value $-\frac{E_{\alpha }}{\mathrm{Rn}}$. The Coats–Redfern method is usually calculated in combination with other methods. The fitting plots for TBPTMH and the addition of H_2_SO_4_ and NaOH are displayed in Figure 7a–c.

From Figure 7, it can be calculated that the E_a_ of TBPTMH and the addition of H_2_SO_4_ and NaOH are 138.61, 113.40, and 118.72 kJ/mol, respectively, and the reaction orders are 1.16, 0.94, and 0.94, individually. Each n value is close to 1. The mechanism functions with n around 1 were selected from Table 2, and the obtained E_a_ was compared with that calculated by the Starink method. The mechanism function with high E_a_ and the great correlation coefficient was selected. Different G(α) from Table 2 were selected to bring into the calculation, and the results are shown in Table 8, Table 9 and Table 10.

Comparing the E_a_ results in Table 8, Table 9 and Table 10 with that calculated by the Starink method, it is found that the E_a_ calculated by the No. 16 mechanism function were the most similar. The difference of each E_a_ was < 10%, and the 10 correlation coefficients were all above 0.94, indicating the reliability of the results. The mechanism functions of TBPTMH and the addition of H_2_SO_4_ and NaOH are G(α)=−ln(1−α); the decomposition mechanisms all follow the law of random nucleation and subsequent growth.

### 3.4. Thermal Decomposition Characteristic Parameters Calculation

#### 3.4.1. TMR_ad_ Calculation Results

**Figure 8:**
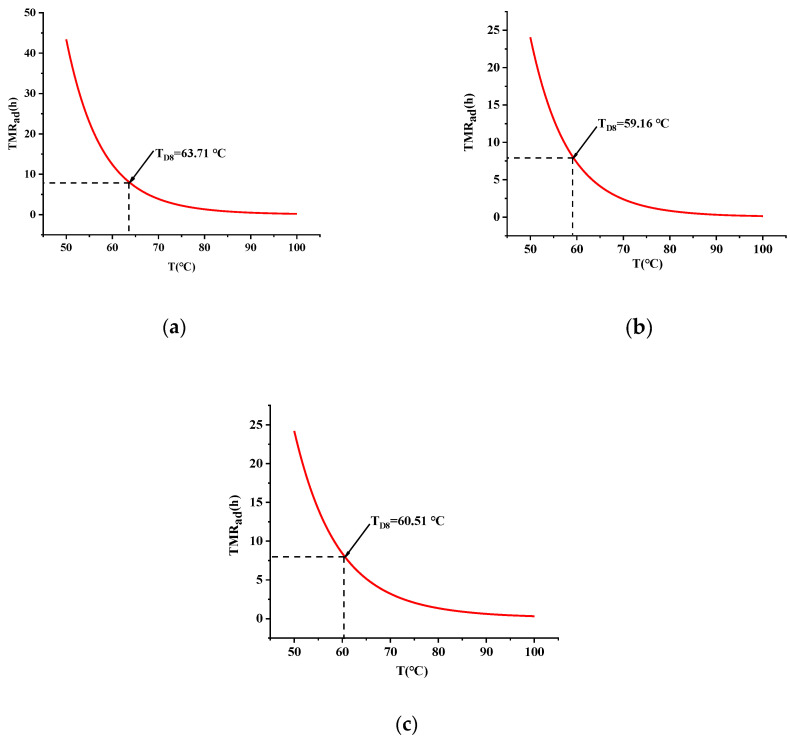
TMR_ad_-temperature curves of TBPTMH (**a**) and mixtures with H_2_SO_4_ and NaOH (**b**,**c**).

A plot of TMR_ad_ versus temperature was calculated according to Equation (5), as shown in Figure 8a–c.

According to the evaluation criteria of the possibility of accidents, T_D8_ (the temperature corresponding to TMR_ad_ = 8 h) was chosen in this paper to simulate the real operating temperature. The T_D8_ of TBPTMH is 63.71 °C, so the temperature should be controlled below 63.71 °C during the actual operation.

The T_D8_ with the addition of H_2_SO_4_ and NaOH are 59.16 °C and 60.51 °C, respectively. Therefore, the T_D8_ ranking of the three systems is: TBPTMH > TBPTMH + NaOH > TBPTMH + H_2_SO_4_. In other words, the effect content of the influencing factor is: NaOH < H_2_SO_4_. H_2_SO_4_ has the most significant effect on the decomposition of TBPTMH. Therefore, the contact hazard of TBPTMH with H_2_SO_4_ is greater compared to NaOH.

#### 3.4.2. SADT Calculation Results

In this section, the SADT values for different packaging mass according to the Semenov model were determined, and the results are presented in Table 11.

From Table 11, the SADT of TBPTMH was 57.58, 55.25, and 52.92 °C for 10, 25, and 50 kg packing masses, respectively. The SADT decreased with increasing packing mass, which is consistent with the literature findings [7].

The SADT of the solution with H_2_SO_4_ and NaOH added are also summarized in Table 11. The comparison results of SADT showed that the SADT difference between the solutions with NaOH added and pure TBPTMH is large regardless of the packing mass, which indicates that the effect of NaOH on the decomposition of TBPTMH under adiabatic conditions is obvious and will advance the value of SADT significantly. Therefore, TBPTMH becomes more dangerous in contact with NaOH.

In general, the relationship between the control temperature and alarm temperature of the sample and SADT is shown in Table 12. Therefore, in this paper, for a package mass of 25 kg, the control temperature and the alarm temperature for pure TBPTMH should be set to 45.25 °C and 50.25 °C, respectively. The control temperature and the alarm temperature for TBPTMH in the presence of H_2_SO_4_ are recommended to be 39.95 °C and 44.95 °C. The control temperature and the alarm temperature of TBPTMH under the action of NaOH are 39.73 °C and 44.73 °C, respectively. Therefore, to ensure safety during production, storage, and usage, the ambient temperature of TBPTMH and the addition of H_2_SO_4_ and NaOH should be controlled below 45, 40, and 40 °C, respectively.

## 4. Conclusions

To better guide the safe storage, transport, and production of TBPTMH, the DSC and Phi-TEC II were used to study the effect of adding H_2_SO_4_ and NaOH on the thermal hazard of TBPTMH. The crucial conclusions are summarized as follows:

The DSC experiments found that the addition of H_2_SO_4_ and NaOH does not change the shape of the exothermic peak, but reduces the T_0_. In an adiabatic environment, the same conclusion can be obtained. The Starink method showed that the E_a_ of TBPTMH and the addition of H_2_SO_4_ and NaOH were 132.49, 116.36, and 118.24 kJ/mol, respectively, and the effect content of the influencing factor was NaOH<H_2_SO_4_. In an adiabatic environment, E_a_ were 129.98, 115.47, and 117.70 kJ/mol, respectively. The mechanism functions of the abovementioned systems were all No. 16: G(α)=−ln(1−α). To ensure safety during the production, storage, and usage, the ambient temperatures of TBPTMH and its mixture with H_2_SO_4_ and NaOH are recommended to be lower than 45, 40, and 40 °C, respectively.

## Figures and Tables

**Table 1:** A brief overview of the literature.

Author	Organic Peroxides	Experimental Equipment	Additives
Yang et al. [7]	TBPTMH	DSC	/
Chen et al. [5]	TBPTMH	DSC	BPO
Tseng et al. [8]	MEKPO	VSP2, DSC	HCl, HNO_3_, H_3_PO_4_, H_2_SO_4_
Liu et al. [2]	CHP, BPO, DCPO	DSC, TAM III, VSP2	H_2_SO_4_, NaOH, Na_2_SO_3_
You et al. [9]	LPO	DSC	HNO_3_

MEKPO—Methylethylketoneperoxide; CHP—Cumene hydroperoxide; BPO—Benzoyl peroxide; DCPO—Dicumyl peroxide; LPO—Dilauroyl peroxide.

**Table 2:** Commonly used thermal decomposition reaction mechanism functions.

No.	G(α)	No.	G(α)	No.	G(α)
1	$\alpha^{2}$	15	${\left[-\ln\left(1-\alpha \right)\right]}^{3/4}$	29	$1-{\left(1-\alpha \right)}^{1/3}$
2	(1−α)ln(1−α)+α	16	−ln(1−α)	30	$3\left[1-{\left(1-\alpha \right)}^{1/3}\right]$
3	${\left[1-{\left(1-\alpha \right)}^{1/2}\right]}^{1/2}$	17	${\left[-\ln\left(1-\alpha \right)\right]}^{3/2}$	31	$1-{\left(1-\alpha \right)}^{1/2}$
4	${\left[1-{\left(1-\alpha \right)}^{1/2}\right]}^{2}$	18	${\left[-\ln\left(1-\alpha \right)\right]}^{2}$	32	$2\left[1-{\left(1-\alpha \right)}^{1/2}\right]$
5	${\left[1-{\left(1-\alpha \right)}^{1/3}\right]}^{1/2}$	19	${\left[-\ln\left(1-\alpha \right)\right]}^{3}$	33	$1-{\left(1-\alpha \right)}^{2}$
6	${\left[1-{\left(1-\alpha \right)}^{1/3}\right]}^{2}$	20	${\left[-\ln\left(1-\alpha \right)\right]}^{4}$	34	$1-{\left(1-\alpha \right)}^{3}$
7	$\left(1-2/3\alpha \right)-{\left(1-\alpha \right)}^{2/3}$	21	ln[α/(1−α)]	35	$1-{\left(1-\alpha \right)}^{4}$
8	${\left[{\left(1+\alpha \right)}^{1/3}-1\right]}^{2}$	22	$\alpha^{1/4}$	36	${\left(1-\alpha \right)}^{-1}$
9	${\left[{\left(1-\alpha \right)}^{-1/3}-1\right]}^{2}$	23	$\alpha^{1/3}$	37	${\left(1-\alpha \right)}^{-1}-1$
10	${\left[-\ln\left(1-\alpha \right)\right]}^{1/4}$	24	$\alpha^{1/2}$	38	${\left(1-\alpha \right)}^{-1/2}$
11	${\left[-\ln\left(1-\alpha \right)\right]}^{1/3}$	25	α	39	lnα
12	${\left[-\ln\left(1-\alpha \right)\right]}^{2/5}$	26	$\alpha^{3/2}$	40	${\ln\alpha }^{2}$
13	${\left[-\ln\left(1-\alpha \right)\right]}^{1/2}$	27	$\alpha^{2}$	/	/
14	${\left[-\ln\left(1-\alpha \right)\right]}^{2/3}$	28	$1-{\left(1-\alpha \right)}^{1/4}$	/	/

**Table 3:** DSC characteristic parameter values.

Sample	β	Mass (mg)	T_0_ (°C)	T_p_ (°C)	T_end_ (°C)	ΔH (J/g)
TBPTMH	2	1.48	109.39	135.03	151.40	741.75
	4	1.45	113.75	141.40	161.53	600.77
	6	1.45	118.32	145.10	168.00	743.98
	8	1.46	124.42	155.89	177.83	609.52
	10	1.52	125.67	155.78	178.07	741.08
TBPTMH + H_2_SO_4_	2	1.46	88.80	137.74	172.62	792.76
	4	1.54	112.74	145.51	165.77	774.44
	6	1.50	119.02	150.44	172.12	804.83
	8	1.48	120.23	154.63	175.57	672.57
	10	1.52	121.89	156.07	176.98	712.87
TBPTMH + NaOH	2	1.48	111.41	139.13	141.62	677.38
	4	1.52	114.48	147.30	167.55	542.19
	6	1.52	118.16	150.48	171.48	715.07
	8	1.53	122.36	155.33	177.75	666.46
	10	1.49	122.91	156.47	179.56	831.12

**Table 4:** Thermal decomposition characteristic parameters of TBPTMH.

Sample	T_0_ (°C)	T_p_ (°C)	ΔT_ad_ (°C)	(dT/dt)max (°C/min)	(dP/dt)max (bar/min)	P_max_ (bar)
TBPTMH	87.68	242.1	154.42	167.9	137.93	32.3
TBPTMH + H_2_SO_4_	81.58	191.24	109.66	61.77	49.21	24.37
TBPTMH + NaOH	83.20	253.98	170.78	192.85	168.36	31.43

**Table 5:** Calculation results by Starink method.

E_a_ (kJ/mol)	α									
	0.1	0.2	0.3	0.4	0.5	0.6	0.7	0.8	0.9	Average
TBPTMH	126.79	132.77	134.86	136.61	136.28	135.21	133.57	131.10	125.22	132.49
TBPTMH + H_2_SO_4_	122.60	117.84	116.22	115.72	116.26	115.53	115.41	114.52	113.12	116.36
TBPTMH + NaOH	128.47	121.45	119.04	118.28	117.46	116.94	116.12	114.74	111.71	118.24

**Table 6:** Corrected adiabatic data of TBPTMH.

Sample	ϕ	T_0_ (°C)	T_p_ (°C)	ΔT_ad_ (°C)	dT/dt_max_ (°C/min)	dP/dt_max_ (bar/min)
TBPTMH	4.23	76.06	577.14	653.20	710.22	583.44
TBPTMH + H_2_SO_4_	4.22	69.00	393.77	462.77	260.67	207.67
TBPTMH + NaOH	4.22	70.68	650.01	720.69	813.83	710.48

**Table 7:** Adiabatic dynamics calculation results.

Sample	E_a_ (kJ/mol)	A (1/s)	n
TBPTMH	129.98	4.92 × 10^16^	2.6
TBPTMH + H_2_SO_4_	115.47	8.73 × 10^13^	1
TBPTMH + NaOH	117.70	4.74 × 10^14^	2.3

**Table 8:** Calculated results of TBPTMH at different G(α) by Coats–Redfern method.

No.	E_a_ (kJ/mol)	R^2^	No.	E_a_ (kJ/mol)	R^2^
1	164.22	0.9518	17	182.29	0.9952
2	186.10	0.9702	18	245.47	0.9953
13	56.21	0.9943	25	78.67	0.9479
15	87.75	0.9948	27	164.22	0.9518
16	119.30	0.9950	31	96.89	0.9784

**Table 9:** Calculated results of TBPTMH with H_2_SO_4_ at different G(α) by Coats–Redfern method.

No.	E_a_ (kJ/mol)	R^2^	No.	E_a_ (kJ/mol)	R^2^
2	185.17	0.9696	16	118.75	0.9951
3	44.70	0.9744	17	181.60	0.9953
13	55.90	0.9944	25	78.22	0.9469
14	76.85	0.9948	26	120.80	0.9497
15	87.33	0.9949	37	162.59	0.9854

**Table 10:** Calculated results of TBPTMH with NaOH at different G(α) by Coats–Redfern method.

No.	E_a_ (kJ/mol)	R^2^	No.	E_a_ (kJ/mol)	R^2^
2	186.79	0.9729	16	119.66	0.9963
3	45.13	0.9778	17	182.96	0.9965
13	56.35	0.9958	25	78.97	0.9514
14	77.46	0.9961	26	121.94	0.9539
15	88.01	0.9962	37	166.37	0.9866

**Table 11:** SADT calculation results.

Sample	Packing Mass (kg)	T_NR_ (°C)	SADT (°C)
TBPTMH	10	64.89	57.58
	25	62.45	55.25
	50	60.02	52.92
TBPTMH + H_2_SO_4_	10	60.00	52.01
	25	57.84	49.95
	50	56.49	48.67
TBPTMH + NaOH	10	59.81	51.98
	25	57.45	49.73
	50	55.53	47.90

**Table 12:** Relationship between control temperature, alarm temperature, and SADT.

SADT (°C)	Control Temperature (°C)	Alarm Temperature (°C)
SADT < 20	<20	SADT-10
20 ≤ SADT ≤ 35	SADT-15	SADT-10
SADT > 35	SADT-10	SADT-5

## Data Availability

The data presented in this study are available on request from the corresponding author.

## Abbreviations

A	Pre-exponential factor (s^−1^)
β	Heating rate (°C/min)
C_vb_	Specific heat capacity of the sample ball (J g^−^^1^ K^−^^1^)
C_vs_	Specific heat capacity of the sample (J g^−^^1^ K^−^^1^)
DSC	Differential scanning calorimetry
(dT/dt)max	Maximum temperature rise rate (°C min^−1^)
(dP/dt)max	Maximum pressure rise rate (bar min^−1^)
E_a_	Activation energy (kJ mol^−^^1^)
G(α)	Denotes the mechanism
ΔH	Heat of decomposition
k	Rate constant
M_b_	Mass of the sample ball (g)
M_s_	Mass of the sample (g)
Phi-TEC II	Adiabatic calorimetry
P_max_	Maximum pressure (bar)
R	Gas constant (8.314 J mol^−^^1^ K^−^^1^)
S	Surface area of packaging (m^2^)
SADT	Self-accelerated decomposition temperature (°C)
T	Reaction temperature (°C)
∆Tad	The adiabatic temperature rise (°C)
T_end_	Ending temperature (°C)
T_f_	The maximum decomposition temperature under adiabatic conditions (°C)
TMR_ad_	Time to maximum rate under adiabatic conditions (h)
T_NR_	The critical non-return temperature (°C)
T_P_	Peak temperature (°C)
U	Surface heat transfer coefficient (J m^−2^ K^−1^)
∅	Thermal inertia factor
